# The effectiveness and safety of cervus and cucumis polypeptide injection in promoting fracture healing after bone fracture surgeries: Protocol for a systematic review and meta-analysis

**DOI:** 10.1097/MD.0000000000014571

**Published:** 2019-02-15

**Authors:** Xia Yang, Kecheng Niu, Xiaoyan Zhang, Baiqing Gao, Bo Feng, Ha Si

**Affiliations:** a Department of Pharmacy; b Department of Hand, Foot and Ankie Surgery; c Department of Orthopaedics, Inner Mongolia Baogang Hospital, The Third Affiliated Hospital of Inner Mongolia Medical University, Baotou, Inner Mongolia Autonomous Region, China.

**Keywords:** cervus and cucumis polypeptide injection, meta-analysis, postoperative fracture, protocol for a systematic review

## Abstract

**Background:**

Bone fractures are a common occurrence, and, according to clinical investigations, approximately 5% to 10% of patients with fractures will suffer from delayed healing or even non-healing. The high efficacy of traditional Chinese medicine in promoting fracture healing has been fully verified over a long history of diagnosis and treatment. Traditional Chinese medicine has a long history of applying Chinese herbs to treat fractures. Cervus and cucumis polypeptide injection has been widely used to promote fracture healing after fracture surgery in clinic, but its efficacy and safety are controversial. For the above reasons, the purpose of this study is to systematically evaluate the efficacy and safety of cervus and cucumis polypeptide injection in promoting fracture healing after bone fracture surgeries and to provide a th**e**oretical basis for the selection of appropriate treatment measures for delayed healing of patients with fractures.

**Methods:**

A total of 8 databases were searched, including the non-Chinese-language databases PubMed, The Cochrane Library, Web of Science, and Embase and the Chinese databases Chongqing VIP Chinese Journal Service Platform (VIP), Wanfang Data Knowledge Service Platform (Wanfang Data), SinoMed and Chinese National Knowledge Infrastructure (CNKI). The databases were queried for publicly released randomized controlled trials of the effectiveness and safety of Cervus and Cucumis polypeptide injection for fracture healing after surgical treatment, and no language restrictions were imposed. The software Review Manager 5.3 was used to evaluate the quality of the selected documents, and Stata 12.0 software was used for statistical analysis.

**Results:**

This review will be to assess the efficacy and safety of cervus and cucumis polypeptide injection in promoting fracture healing after bone fracture surgeries.

**Conclusion:**

Our study will use systematic evaluation to objectively evaluate the efficacy and safety of cervus and cucumis polypeptide injection in promoting fracture healing after fracture surgery. It will provide theoretical basis for guiding clinical practice and benefit more patients.

**Ethics and dissemination:**

This study is a systematic review that does not require ethical approval and meets the requirements of protocol for a systematic review and meta-analysis. At the same time, this study does not involve the recruitment of patients. All data are from published academic papers.

**Protocol and registration:**

A protocol had been registered for this systematic review and meta-analysis in PROSPERO. (registration number: CRD42019120965).

## 1 Introduction

### 1.1 Current status of delayed Union and nonunion of fracture

Bone fractures are a common occurrence, and, according to clinical investigations, approximately 5%- to 10% of patients with fractures will suffer from delayed healing or even non-healing.^[1]^ With the advancement of modern medicine, various treatment methods are now available for fractures. Fixation and surgical methods are rapidly progressing. Internal fixation is increasingly reliable, and surgical techniques are increasingly successful, but the healing rate of bone fractures has failed to change significantly.^[2]^ Fracture healing is a complex biological process involving the phases of blood circulation, bone renewal, and osteogenic calcification, progressing through the main stages of the granulation tissue, callus formation and bone remodelling.^[3,4]^ According to traditional estimates, tendon or bone injury lasts approximately 100 days; if true, this assumption explains the slow rate of fracture healing. Many growth factors accelerate the repair of fractures through different roles including cell proliferation, differentiation and calcification in different stages of fracture repair. Clinically, the treatment of bone fractures is based mainly on manual or open reduction, fixation and functional exercise. Most patients suffer from pain and swelling of the affected limbs after treatment or surgery. Therefore, recovery after restoration is a focus of clinical treatment. Various medicines are commonly used in clinical practice to promote recovery after initial treatment, but the effects of most such medications are not very satisfactory.

### 1.2 Description of the intervention

The high efficacy of traditional Chinese medicine in promoting fracture healing has been fully verified over a long history of diagnosis and treatment. Traditional Chinese medicine has a long history of applying Chinese herbs to treat fractures.^[5,6]^ Clinical practice has demonstrated that Chinese medicine provides unique advantages in the treatment of fracture healing; the concepts of integrity, syndrome differentiation, and the combination of motion and stillness are followed.^[7]^ The medicine selected in this study, Cervus and cucumis polypeptide injection (CCPI), is a compound preparation consisting of a sterile aqueous solution of extracts from skeletons of *Cervus nippon Temminck and dry*, mature seeds from *Cucumis melo L*.

The main active components of CCPI are bone-growth-inducing polypeptide biological factors and free amino acids and organic calcium and phosphorus from melon seed extract. The functional effects of this product include regulating bone metabolism, stimulating osteoblast proliferation, promoting the formation of new bone, regulating calcium and phosphorus metabolism, increasing the deposition of bone calcium, preventing osteoporosis and providing anti-inflammatory analgesia and anti-rheumatism. At present, CCPI is used in the treatment of various medical conditions in China, including various types of fractures, delayed fracture healing, osteoarthritis of the knee and other joints, rheumatoid arthritis and other conditions, achieving great clinical efficacy.^[8]^ Despite this successful use, we still have no clear understanding of the mechanism of action or the exact degree of efficacy of CCPI. Therefore, domestic scholars have continued to study the mechanism of action and the efficacy of the medicine. For the above reasons, the efficacy and safety of CCPI in promoting fracture healing after surgical repair are systematically evaluated in this paper, and a reference for the treatment of delayed postoperative healing of fractures is provided.

## 2 Data and methods

### 2.1 Protocol and registration

A protocol had been registered for this systematic review and meta-analysis in PROSPERO (registration number: CRD42019120965).

### 2.2 Eligibility criteria

#### 2.2.1 Types of studies

Randomized controlled trials of CCPI as a way to promote fracture healing after surgery were examined. There were no restrictions on language, and studies were included regardless of whether blinding was used to reduce publication bias.

#### 2.2.2 Types of participants

All selected patients must meet the following criteria:

1. All the patients met the relevant diagnostic criteria for limb fractures in *Practical Orthopaedics*;^[9]^
2. all the patients were treated with surgical open reduction and internal fixation or intramedullary nail fixation; and
3. none of the patients suffered from diseases of the heart, liver, brain, kidney or other important organs.

No limitations were imposed on the basis of age, gender, case source, course of recover, or fracture site.

#### 2.2.3 Ethics approval

This study is a systematic review. All patient data are obtained from published papers.

#### 2.2.4 Types of interventions

The patients in the experimental group were subject to CCPI + conventional anti-infective treatment (CAIT) (recorded as the dosage, time and frequency of medication and the course of treatment); the patients in the control group were subject to conventional anti-infective treatment (CAIT) (recorded as the dosage, time and frequency of medication and the course of treatment).

#### 2.2.5 Types of outcome measures

##### 2.2.5.1 Primary outcomes

(1) The healing times of the following types of fractures were examined: [(I) humeral shaft fracture; (II) femoral shaft fracture; (III) humerus fracture; (IV) patella fracture and 2 fractures of humerus; (V) ulna fractures]; (2) clinical efficacy.

Efficacy was assessed by comparing healing times between fractures treated with CCPI + CAIT and those treated with CAIT alone. The healing times of CCPI-treated and control-treated fractures were compared, and the healing time of the CCPI group was classified as more than 1/3 shorter, 1/3 to 1/4 shorter, 1/4 to 1/5 shorter, or not substantially shorter than the healing time of the control group.

The clinical healing assessment criteria were as follows:^[10]^

1. elimination of local oedema;
2. disappearance of local tenderness;
3. disappearance of abnormal activity of the fracture;
4. after external fixation, the injured limb is able to lift a 1 kg object within 1 minute in the case of an upper limb, or the patient can walk at least 30 steps on flat ground with the injured limb within 3 minute in the case of a lower limb;
5. the fracture site exhibits no deformation in 2 weeks of consecutive observation;
6. the X-ray results show that the epiphysis has a fusiform connection with uniform density;
7. the fracture line is vague.

The first day on which the patient was observed to reach the above criteria was considered the date of clinical healing.

##### 2.2.5.2 Secondary outcomes

1. the other indicators consisted of the occurrence rate of calluses;
2. the regression rate of swelling;
3. the visual analogue scale (VAS) scores of postoperative 7-day pain intensity;
4. incidence of adverse reactions.

The VAS was used on the 3rd, 7th and 14th days after fracture repair surgery to evaluate the pain intensity at the surgical site.^[11]^ That is, a straight line with a length of 10 cm was drawn on paper. The numbers “0” and “10” were written at opposite ends. A score of “0” represented no pain, and “10” represented the most intense pain; a score less than “4” represented mild pain, a score of 4 to 7 represented moderate pain, and a score greater than 7 represented severe pain. The patient was asked mark the corresponding position on the straight line according to the intensity of pain that he/she felt, and then the distance from the starting point to the marked point (in cm) was measured with a ruler to score the response. The higher the score, the higher the intensity of pain being represented.

The degree of swelling of the affected limbs after fracture surgery was assessed and recorded on the 1st, 3rd, and 7th days.^[12]^ The criteria for swelling assessment were as follows. Grade I: the skin of the affected limb was tighter than normal skin with dermatoglyphs; Grade II: the skin of affected limb was tighter than normal skin without dermatoglyphs, and the skin temperature was slightly elevated, with tension blisters appearing; Grade III: the skin of the affected limb was bright, the striae had disappeared, the skin temperature was obviously increased, and tension blisters were present.

### 2.3 Search methods for the identification of studies

#### 2.3.1 Electronic searches

The contents of several online databases, including the non-Chinese-language databases PubMed, Cochrane Library, Web of Science, and Embase and the Chinese databases Chongqing VIP, Wanfang Data, SinoMed, and Chinese National Knowledge Infrastructure (CNKI), were retrieved by computer. The search terms include a combination of keywords and free words. The retrieval languages were not limited.

#### 2.3.2 Other sources

Non-electronic papers and periodicals after 2010 were retrieved manually. Additionally, after relevant studies were identified through database searches, their reference sections were examined to guarantee that as many relevant documents as possible were collected.

#### 2.3.3 Search strategy

The Chinese search terms consisted of (“Luguaduotai” or “Mianshu” or “Songmeile”) and (“Guzhe” or “Guyuhe” or “Gubuyuhe” or “Yanchiyuhe”); the English search terms consisted of (“Luguaduotai” or “Mianshu” or “Songmeile” or “Cervus and Cucumis Polypeptide”) and (“Fracture Healing[Mesh]” or “Fractures, Ununited[Mesh]” or “Fracture Healings” or “Healing, Fracture” or “Healings, Fracture” or “Fracture, Ununited” or “Ununited Fracture” or “Ununited Fractures” or “Delayed union”), etc. The Search strategy are shown in Table 1.

**Table 1:**
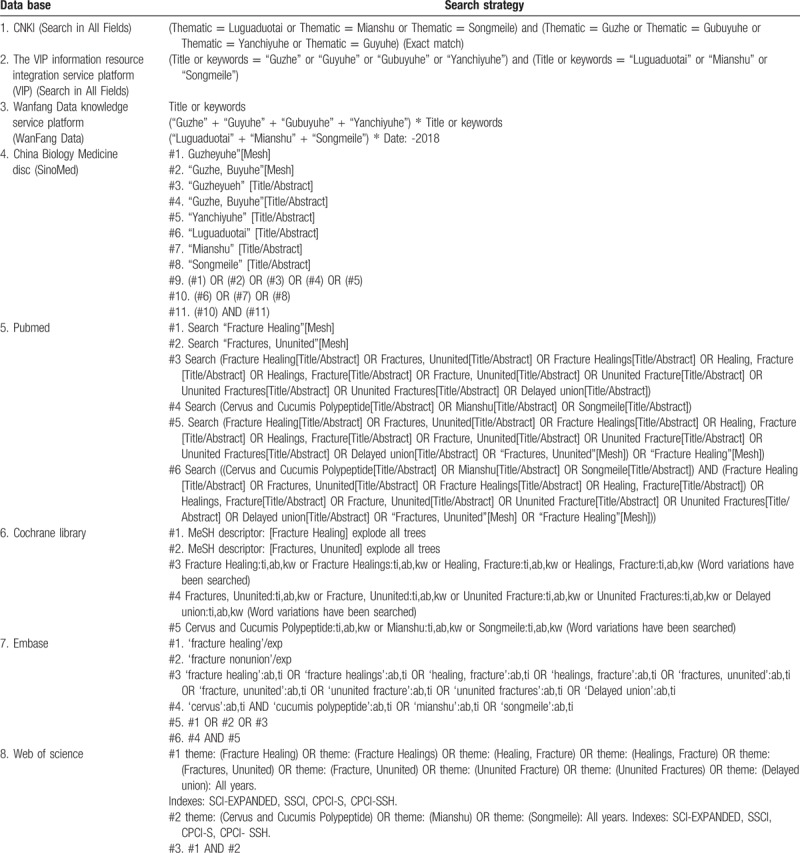
Search strategy.

### 2.4 Data collection and analysis

#### 2.4.1 Data extraction

**Figure 1:**
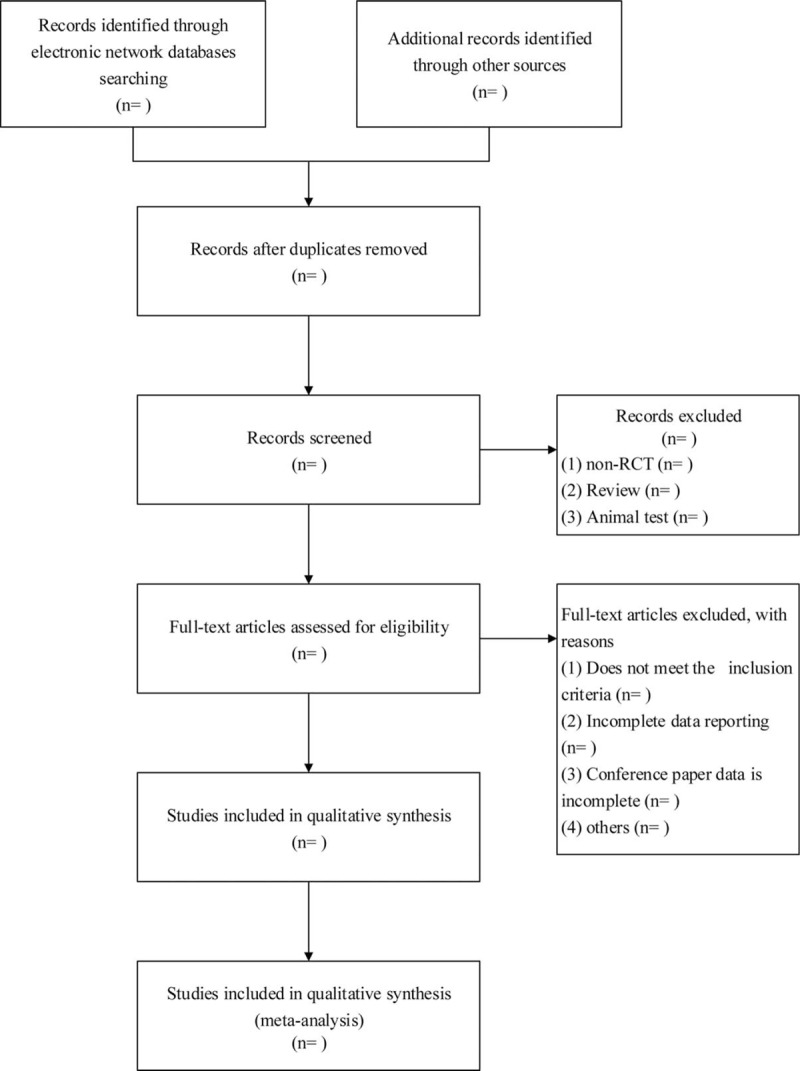
Flow diagram for study selection process used in this meta-analysis.

Data extraction is accomplished independently by 2 members of the research group. The data extracted from each study included the author and time of publication; randomization method; basic data of studied subjects such as the number of participants, loss to follow-up, specific age, gender, and male-female ratio; interventions; drug dosage; course of treatment; outcome observation indicators; and availability of adverse events. Two independent reviewers applied pre-defined criteria to select appropriate articles. Any disputes between the reviewers were resolved via mutual discussion or with the assistance of a third party. The flow chart of study selection is shown in Figure 1.

#### 2.4.2 Addressing missing data or unclear measurement scales

For papers with incomplete data coverage, the author of the paper is first requested by e-mail or telephone. If the author of the paper does not agree to provide data or cannot contact the author of the paper, we will discuss the trade-offs of the paper.

#### 2.4.3 Assessment of heterogeneity

The assessment of heterogeneity is completed independently by 2 members of the research team. Data extraction is accomplished independently by 2 members of the research group. The quality evaluation was included in the risk of bias in each study based on the RCT risk assessment tool recommended by the Cochrane Handbook for Systematic Reviews of Interventions 5.1.0, including 7 items addressing the following aspects:^[13]^

1. Selection bias, including the generation, distribution and concealment of random sequences;
2. implementation bias, that is, whether blinding was applied to implementers and subjects;
3. measurement bias, that is, blinding for outcome assessment;
4. bias through loss to follow-up, that is, completeness or incompleteness of outcome data;
5. publication bias, that is, availability of selected reports and findings; and
6. other biases. According to the evaluation criteria, the seven-item scale was used to evaluate studies as “high risk of bias (H)”, “low risk of bias (L)” or “unknown risk of bias (U)”.

These criteria were applied by 2 evaluators, whose scores were then compared; if the results were inconsistent, the disagreement was resolved through discussion or consultation with a third researcher.

#### 2.4.4 Data analysis

Meta-analysis: Statistical analysis was performed by using the software Stata 12.0 for meta-analysis.

1. Selective effect size: If the index of literature results included in the test is a binary variable, then the statistics of the efficacy analysis may be represented with the relative risk and 95% confidence interval; the mean difference and its 95% confidence interval are used to represent continuity changes.
2. Test heterogeneity: Statistical homogeneity testing was applied to test the degrees of variation degrees of the original research results and clearly incorporate them into the homogeneity of the test.
3. Meta-analysis: According to the results of the heterogeneity test, when *P* ≥ .05 and I^2^ < 50 are satisfied, the consistency of the results is high, and a fixed effect model may be applied. If *P* < .05 and I^2^ ≥ 50 are satisfied, meaning that the heterogeneity of results should not be ignored, if clinical significance of the included studies still exists, then the random effect model is used.

#### 2.4.5 Sensitivity analysis

A sensitivity analysis was conducted by considering the following different situations and comparing the results to those of the original meta-analysis to examine the stability of the analysis results. If the analysis result are consistent with the original results, the original results are considered credible; if the sensitivity analysis results are inconsistent with the original results, the original results are considered unstable, and there may be important factors [(1) Fixed effect model and random effect model; (2) Different estimation methods for missing data; (3) Elimination of extreme research cases (extreme effect, very small sample size, extremely high loss rate, etc.) or not; (4) Elimination of the case with low quality research] affecting the effects of the treatment measures. The reliability of the conclusion is poor, and the source of the discrepancy must be determined, analysed, and explained.

#### 2.4.6 Subgroup analysis and solutions to heterogeneity

In the analysis of the primary and secondary outcomes, if there is a large heterogeneity in the results, after the random factors are excluded, based on the data provided in the literature, factors that may differ in the included studies, such as case characteristics, intervention measures, duration of intervention, study area, etc. The included studies were divided into 2 or more groups according to one of the above factors to observe whether the difference between the effect sizes of each sub-combination and the effect was statistically significant. That is, whether there is an interaction between the sub-combination effect quantity and the grouping factor (interaction), thereby judging whether the grouping factor is heterogeneous between the results of the studies.

#### 2.4.7 Assessment of reporting bias

Publication bias is caused by the increased probability of acceptance and publication by journals of positive data contained in papers implying statistical significance, and this form of bias is difficult to control. The funnel plot technique is commonly used to detect publication bias. The software Stata 12.0 was used in this study. The outcome indicators reported by ≥6 studies with I^2^ ≥ 50 were selected, and Egger test was used to test the publication bias.

### 2.5 Ethics and dissemination

This study is a systematic review that does not require ethical approval. This study has been registered on the PROSPERO website (https://www.crd.york.ac.uk/PROSPERO/#register) and meets the requirements of protocol for a systematic review and meta-analysis. At the same time, this study does not involve the recruitment of patients. All data are from published academic papers.

## 3 Discussion

The possibility of people suffering from trauma rises with the increase in their activity and space, and the incidence of fractures increase with China's approach to an ageing society and an increase in patients with osteoporosis. The fracture healing is a complex physiological process accompanied by local haematoma and inflammation. The healing process involves the proliferation, differentiation and matrix mineralization of osteoblasts. The incidence of fracture nonunion accounts for 5% to 10% of all the fractures. A variety of physical and physiological factors influence the fracture healing.^[14]^ Healing of the fracture occurs 6 to 8 months after the fracture. If the fracture does not heal within 9 months after the operation and no signs of fracture repair are found after 3 consecutive months of X-ray examination, then such fracture be diagnosed as the nonunion.^[15]^ The treatment for nonunion is a challenge for orthopaedic surgeons, as nonunion often means that extremely complicated, lengthy and costly treatment is required.^[16]^ Meanwhile, the nonunion will cause many complications like the loss of function, pain, degenerative joint, etc. Moreover, the patients usually have great psychological barriers due to their inability to work. Traditional Chinese medicine plays a very important role in the treatment of orthopaedic diseases to promote fracture healing and shorten the healing time of fracture.

CCPI is a compound preparation and sterilized aqueous solution consisting of dried and mature seeds of *Cucumismelo L*. and Cucurbitaceae. It mainly contains osteoinductive polypeptide biological factor, melon seed extract, various free amino acids, and organic calcium and phosphorus. Studies have shown that the rabbits subject to intramuscular injection of cucurbit polypeptide in BMP2 and TGF-β1 positive staining cells early,^[17]^ and sooner positive cells with larger dose appear, the more the number will be, indicating that the CCPI can effectively promote the synthesis of local endogenous growth factors in the bone fracture in early period of fracture, including BMP, TGF-β, FGF, etc.^[18,19]^ Under the action of these growth factors, the differentiation of granulation tissue into chondrocytes and osteoblasts is accelerated, and mesenchymal stem cells that are localized to fractures also multiply in the presence of these growth factors and differentiate into osteoblasts to form new bones. Therefore, we chose CCPI, the medicine widely used in China to promote fracture healing, for the purpose of studying its clinical efficacy and safety and providing evidence for evidence-based medicine for clinicians.

## Author contributions

Ha Si is the proponent of this research concept. Xia Yang, Kecheng Niu and Bo Feng are responsible for the collection of the literature. Ha Si is responsible for the application of the research fund. Xiaoyan Zhang and Baiqing Gao independently conducted the screening and data of the literature. The extraction, Xia Yang and Kecheng Niu were responsible for the use of statistical software, and all the authors participated in the writing of the paper. Ha Si conducted a final review of the paper.

**Conceptualization:** Ha Si.

**Data curation:** Xia Yang, Kecheng Niu, Bo Feng.

**Formal analysis:** Xiaoyan Zhang, Baiqing Gao.

**Methodology:** Xia Yang, Kecheng Niu.

**Software:** Xia Yang, Kecheng Niu.

**Supervision:** Ha Si.

**Writing – original draft:** Xia Yang, Kecheng Niu, Xiaoyan Zhang, Baiqing Gao, Bo Feng.

**Writing – review:** Ha Si.

Ha Si orcid: 0000-0003-0724-9418.
